# Introducing oriented Laplacian diffusion into a variational decomposition model

**DOI:** 10.1186/s13634-016-0415-2

**Published:** 2016-11-10

**Authors:** Reza Shahidi, Cecilia Moloney

**Affiliations:** Department of Electrical and Computer Engineering, Memorial University of Newfoundland, Prince Philip Avenue, St, John’s, Canada

**Keywords:** Image decomposition, Variational methods, Image processing, Osher-Solé-Vese model, Oriented Laplacian, Image denoising

## Abstract

The decomposition model proposed by Osher, Solé and Vese in 2003 (the OSV model) is known for its good denoising performance. This performance has been found to be due to its higher weighting of lower image frequencies in the *H* ^−1^-norm modeling the noise component in the model. However, the OSV model tends to also move high-frequency texture into this noise component. Diffusion with an oriented Laplacian for oriented texture is introduced in this paper, in lieu of the usual Laplacian operator used to solve the OSV model, thereby significantly reducing the presence of such texture in the noise component. Results obtained from the proposed oriented Laplacian model for test images with oriented texture are given, and compared to those from the OSV model as well as the Mean Curvature model (MCM). In general, the proposed oriented Laplacian model yields higher signal-to-noise ratios and visually superior denoising results than either the OSV or the MCM models. We also compare the proposed method to a non-local means model and find that although the proposed method generally yields slightly lower signal-to-noise ratios, it generally gives results of better perceptual visual quality.

## Introduction

### Image decomposition

According to the definition of Meyer [1], image decomposition is the process of splitting an image into components of different priorities, usually so that the original image is roughly equal to the sum of these components. Often the original image is denoted by *f*, which is considered to approximately equal the sum of two components, *u* and *v*, i.e. *f*≈*u*+*v*. In Meyer’s 2001 monograph [1], decomposition was deemed to be important for image coding and transmission. In addition to the problem of decomposition being interesting and important in its own right [2], decomposition has also proven to be useful for texture discrimination [3], image denoising [4], image inpainting [5] and image registration [6]. In this paper, the focus is on the application of decomposition to the problem of the denoising of images with oriented texture.

### Meyer’s decomposition framework

Meyer’s decomposition framework models the various components of the decomposition as having small norms in different Banach spaces, i.e. complete normed vector spaces. In practical implementations of this framework [3], the energy method is used, in which an energy is defined over the image being decomposed and its components, and is subsequently minimized using the Calculus of Variations.

The first method that could be considered to fall under the image decomposition framework is the total variation model of Rudin, Osher and Fatemi [7]. The energy for the total variation model is defined as

1 $$ \mathbf{E_{TV}}(u)=\int_{\Omega}|\nabla u|dxdy+\lambda\int_{\Omega}(f-u)^{2}dxdy,  $$

where the original image is *f* and *f*≈*u*+*v*. The first term $\left (\int _{\Omega }|\nabla u|dxdy\right)$ is included to produce a bounded variation (piecewise-smooth) image *u* upon energy minimization, while the second term is a fidelity term, which ensures that the final *u* component is close to the initial image *f*. Although the minimization of *E*_*TV*_(*u*) preserves sharp edges, it destroys fine structure such as texture. However, Eq. 1 has been used successfully for the denoising and deblurring of images of bounded variation.

In [1], Meyer proposed modifying the second term in the above energy of Eq. 1 by changing the *L* ^2^-norm of the residual *v* to the ∗-norm of this residual, where ∗ is a norm on a suitably defined Banach space *G*. The ∗-norm on *G* is defined by

2 $$ \Vert v\Vert_{\ast}=\inf_{g_{1},g_{2}}\left\Vert \sqrt{{g_{1}^{2}} (x,y)+{g_{2}^{2}}(x,y)}\right\Vert_{L^{\infty}},  $$

over all *g*_1_ and *g*_2_ such that $v=\text {div}({\vec {g}})$ where ${\vec {g}}=(g_{1},g_{2})$.

### The Osher-Solé-Vese decomposition model

A second attempt at minimizing the ∗-norm of Eq. 2 was made by Osher, Solé and Vese in [4]. They used the Hodge decomposition of $\vec {g}$, which splits $\vec {g}$ into the sum of the divergence of a single valued function and a divergence-free vector field. The energy functional for their model is

3 $$ \mathbf{E_{OSV}}(u) = \int_{\Omega}|\nabla u| dxdy + \lambda \int_{\Omega}\left|\nabla\left(\Delta^{-1}\right)(f-u)\right|^{2} dxdy  $$

The PDE that they obtained as a result of minimization of the above functional is

4 $$ u_{t} = -\frac{1}{2\lambda}\Delta \left[\text{div}\left(\frac{\nabla u}{|\nabla u|}\right) \right]-(u-f)  $$

with adiabatic boundary conditions. This equation was found by approximating $\vec {g}$ as having no divergence-free part, and approximating the *L* ^*∞*^ part of the ∗-norm in Eq. 2 with the square of the *L* ^2^-norm of the same function. The resulting energy functional includes an inverse Laplacian of *f*−*u*; this was eliminated in [4] by showing that under some rather relaxed conditions, found in the second Remark of that paper, the gradient descent solution of an Euler-Lagrange equation *u*_*t*_=−*E* *OSV*′(*u*), where *E* *OSV*′(*u*) is the first variation of *E*_*OSV*_ with respect to *u*, converges to the same solution as the equation *u*_*t*_=*Δ* *E* *OSV*′(*u*). Thus no inverse Laplacian appears in Eq. 4. Additionally, the auxiliary functions *g*_1_ and *g*_2_ of Eq. 2 are no longer involved in the PDE of Eq. 4, as they disappear in the derivation.

## Incorporating oriented diffusion into the OSV model

### Motivation and initial derivation

If we denote the curvature of the level lines of the cartoon component *u* by $K(u) = \text {div}\left (\frac {\nabla u}{|\nabla u|}\right)$, Eq. 4 becomes

5 $$ u_{t} = -\frac{1}{2\lambda}\Delta K(u) -(u-f).  $$

Observe that Eq. 5 consists of two terms, one with the negative Laplacian of the curvature of the level lines of *u*; the other a fidelity term, which makes sure that the evolved image does not stray too far away from the original image *f*. Equation 5 has been shown to perform well for denoising [4]. Since the original image *f* is noisy, and the second term keeps the evolved image close to this noisy image, the denoising itself must be due to the first term; i.e. the negative Laplacian term.

Attention is now restricted to images with oriented texture. With this restricted focus, instead of including the Laplacian of the curvature, *Δ* *K*(*u*), as in Eq. 5, the oriented Laplacian [8] of the curvature can be taken instead. The new equation becomes

6 $$ u_{t} = -\frac{1}{2\lambda}\left(c_{\zeta}K_{\zeta\zeta} + c_{\eta}K_{\eta\eta}\right) - (u-f)  $$

**Fig. 1:**
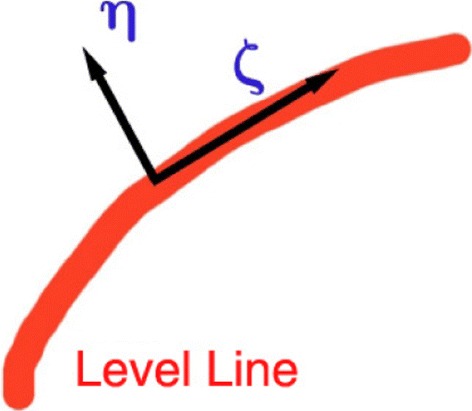
Illustration of isophote and gradient directions

Here *ζ* is the isophote direction of the curvature in the regions of the image where there is a dominant orientation, and *η* is the gradient direction of the curvature in such regions. An illustration of these directions is shown in Fig. 1.

There are also two conductivity coefficients *c*_*ζ*_ and *c*_*η*_ which determine how much diffusion there is in the isophote and gradient directions respectively.

The values of the second directional derivatives of the curvature in its isophote and gradient directions can be computed without actually calculating the isophote and gradient angles. This is done by using the general formula for the second-order directional derivative of the function *K* in the direction **w**=(*w*_*x*_,*w*_*y*_), i.e.

$$K_{\mathbf{ww}} = {w_{x}^{2}}K_{xx} + 2w_{x}w_{y}K_{xy} + {w_{y}^{2}}K_{yy}, $$

and then substituting the unit gradient and isophote vector directions for **w**. The unit gradient vector is simply

$$\mathbf{w_{grad}} = \left(\frac{K_{x}}{\sqrt{{K_{x}^{2}}+{K_{y}^{2}}}},\frac{K_{y}}{\sqrt{{K_{x}^{2}}+{K_{y}^{2}}}}\right), $$

and the unit isophote vector is

$$\mathbf{w_{iso}} = \left(\frac{-K_{y}}{\sqrt{{K_{x}^{2}}+{K_{y}^{2}}}},\frac{K_{x}}{\sqrt{{K_{x}^{2}}+{K_{y}^{2}}}}\right). $$

The formulae for the second directional derivative of the curvature in the isophote direction (*ζ*) and its gradient direction (*η*) are thus

7 $$\begin{array}{@{}rcl@{}} K_{\zeta\zeta} = \frac{-2K_{x}K_{xy}K_{y} + K_{xx}{K_{y}^{2}}+{K_{x}^{2}}K_{yy}}{{K_{x}^{2}}+{K_{y}^{2}}} \end{array} $$

8 $$\begin{array}{@{}rcl@{}} K_{\eta\eta} = \frac{{K_{x}^{2}}K_{xx}+2K_{x}K_{xy}K_{y}+{K_{y}^{2}}K_{yy}}{{K_{x}^{2}}+{K_{y}^{2}}}. \end{array} $$

The expressions for the two directional derivatives in Eqs. 7 and 8 can be substituted into Eq. 6. Recall that the function *K* represents the curvature of the cartoon component *u*.

In our experiments for this paper, better results were obtained in practice by using the normalized isophote orientation estimates of the original image *f* instead of those of the curvature image *K*. An initial explanation for the improvement in results is the sensitivity to noise of the formulae in Eqs. 7 and 8.

We now explain how estimates of the normalized isophote and gradient directions are extracted from the original noisy image using the linear structure tensor (Section 2.2.1), as well as how this image is separated into oriented and non-oriented regions (Section 2.2.2). This separation by region is important to decide whether anisotropic or isotropic diffusion should be performed at each image pixel, and the directional estimates are important to determine which directional diffusion parameters are used for anisotropic diffusion at each pixel in the oriented region. After this explanation in Section 2.2, we return to the derivation of the proposed model in Section 2.3 using these extracted gradient and isophote orientation estimates.

### Orientation and coherence calculation

The proposed algorithm depends on the noise-resistant computation of orientation at each pixel in the image, in addition to the detection of the image regions that are coherent, or roughly consisting of one orientation. Two methods were considered in our experiments by which such calculations could be performed and be robust to noise. Both use the structure tensor, or the outer product of the image gradient with itself. The first is called the linear structure tensor, which corresponds to a linear diffusion or Gaussian blurring of the structure tensor elements, and the second is called the nonlinear structure tensor, an extension of the linear structure tensor which corresponds to nonlinear diffusion of the structure tensor entries. Only the linear structure tensor is described here, since it was found to be more efficient computationally than the nonlinear structure tensor, and gave results of similar quality. The linear structure tensor is now described, along with methods of determining the orientation coherence [9]. If not otherwise specified, it is assumed the orientation refers to the gradient orientation, or the orientation in which the image changes the most in intensity.

#### Linear structure tensor

The structure tensor is defined as the outer product of the image gradient vector with itself. Supposing that the image is *f*, then the structure tensor *J* at each pixel is defined as

$$ J= \left(\begin{array}{cc} {f_{x}^{2}} & f_{x}f_{y} \\ f_{x}f_{y} & {f_{y}^{2}} \end{array}\right). $$

It was found that better denoising results were obtained for the proposed method by using the following derivative filters (see e.g. [10]) to determine *f*_*x*_ and *f*_*y*_

9 $$ D_{x} = \frac{1}{32}\left(\begin{array}{ccc} -3 & 0 & 3\\ -10 & 0 & 10\\ -3 & 0 & 3 \end{array}\right)  $$

and

10 $$ D_{y} = \frac{1}{32} \left(\begin{array}{ccc} 3 & 10 & 3\\ 0 & 0 & 0\\ -3 & -10 & -3 \end{array}\right).  $$

To further increase robustness to noise, the resulting structure tensor *J* is blurred element-wise with a Gaussian filter *G*_*σ*_ of standard deviation *σ*, to obtain *J*_*σ*_=*G*_*σ*_∗*J*. Then the gradient direction at each pixel, *θ*_*i*,*j*_, is calculated to be the angle between the eigenvector $\overrightarrow {w}_{1}$ of *J*_*σ*_ corresponding to its larger eigenvalue *λ*_1_ and the horizontal axis, or more simply, if we consider $\overrightarrow {w}_{1}$ of *J*_*σ*_ to be a vector in the complex plane

$$\theta_{i,j} = {\mathbf{arg}} \overrightarrow{w}_{1}. $$

The isophote vector $\overrightarrow {f_{iso}} = (f_{iso,x},f_{iso,y})$ is computed as the unit vector perpendicular to *θ*_*i*,*j*_, thus at an angle $\frac {\pi }{2}$ radians greater than *θ*_*i*,*j*_ from the horizontal axis; i.e. if we consider the isophote vector as a vector in the complex plane

$${\mathbf{arg~}} \overrightarrow{f_{iso}} = {\mathbf{arg}}\overrightarrow{w}_{1}+\frac{\pi}{2}. $$

#### Orientation coherence and oriented region determination

For the proposed algorithm, it is necessary to separate the image into oriented and non-oriented regions, so that different variational models can be applied to each of the two region types. A region is defined to be non-oriented when its orientation coherence is less than a pre-determined threshold; this coherence function is a measure of how uniform gradient directions are around a pixel. In [11], the coherence of *f* is measured directly using a small window *W* around each pixel by the formula

$$\text{coher}(\theta_{i,j}) = |\nabla f|_{i,j}\frac{\sum_{(u,v) \in W} ||\nabla f|_{u,v}\cos(\theta_{i,j}-\theta_{u,v})|}{\sum_{(u,v) \in W}|\nabla f|_{u,v}}, $$

where *θ*_*i*,*j*_ is the orientation calculated from the linear structure tensor *J* at pixel (*i*,*j*) (which can be found because the tensor determines the gradient vector at each pixel); generally *W* is chosen to be 7 × 7 pixels. In this paper, this formula is used to determine the orientation coherence at each pixel, thus allowing oriented and non-oriented regions to be separated.

The threshold used for the coherence of the orientations of *f* is dependent on the image. Call this parameter cohe*r*_*thresh*_; we set cohe*r*_*thresh*_=15 for all experiments in this paper.

Now define the oriented region to be *Ω*_*O*_, the union of pixels in the image where there is a dominant orientation in *f*. Similarly, we define the non-oriented region to be *Ω*_*NO*_, the union of pixels in the image where there is no such dominant orientation.

### The oriented Laplacian Osher-Solé-Vese model

After Eqs. 7 and 8 in Section 2.1, we stated that using the normalized isophote orientation estimates of the original image yielded better denoising results in our experiments. After this, in Section 2.2 we explained how these isophote orientation estimates were computed using the linear structure tensor. Now using the isophote orientation vectors (*f*_*iso*,*x*_,*f*_*iso*,*y*_) at each pixel, we obtain the following noise-resistant approximation of the second directional derivative of the curvature in the isophote direction

11 $$ K_{\zeta\zeta} \approx f^{2}_{iso,x}K_{xx} + 2f_{iso,x}f_{iso,y}K_{xy} + f^{2}_{iso,y}K_{yy}  $$

which is subsequently substituted in the oriented Laplacian expression in Eq. 6. Note that the denominators in Eqs. 7 and 8 do not appear since we are dealing with the normalized isophote estimates, as opposed to Eqs. 7 and 8, where the partial derivatives of *K* are unnormalized.

For the coefficient *c*_*η*_ in Eq. 6, the usual Perona-Malik diffusivity function

12 $$ c_{\eta} = g(|\nabla K(u)|) = \frac{1}{1+\frac{|\nabla K(u)|^{2}}{{K_{d}^{2}}}},   $$

could be chosen (with *K*_*d*_ set to 10 after experimentation). However, we found that just setting *c*_*η*_ to zero gave similar results and was simpler; consequently the expression in Eq. 12 was not used. With the coefficient *c*_*η*_ set to zero, there is no diffusion or denoising in the image gradient direction. To promote smoothing in the image isophote direction, the coefficient *c*_*ζ*_ is set equal to 1. Therefore Eq. 6 becomes

13 $$  u_{t} = -\frac{1}{2\lambda}K_{\zeta\zeta} - (u-f),  $$

and substituting the approximation of Eq. 11 for *K*_*ζζ*_ yields the final equation for the iterative solution of the proposed decomposition model

14 $$ \begin{aligned} &{}u_{t} = -\frac{1}{2\lambda}\left(f^{2}_{iso,x}K_{xx} + 2f_{iso,x}f_{iso,y}K_{xy} + f^{2}_{iso,y}K_{yy}\right) - (u-f)\\ & {\kern-7.5pt}= -\frac{1}{2\lambda}\left({\zeta_{x}^{2}} K_{xx} + 2\zeta_{x}\zeta_{y} K_{xy} + {\zeta_{y}^{2}} K_{yy}\right) - (u-f), \end{aligned}  $$

where (*ζ*_*x*_,*ζ*_*y*_)=(*f*_*iso*,*x*_,*f*_*iso*,*y*_) is a unit vector defined for each pixel in the image pointing in the isophote direction.

Within the oriented region *Ω*_*O*_, Eq. 14 is used, whereas within the non-oriented region *Ω*_*NO*_, the ordinary evolution equation for Osher-Solé-Vese decomposition, as in Eq. 4 is utilized. Each equation and region involves a weighting parameter *λ*. This parameter becomes *λ*_*O*_ in *Ω*_*O*_, and *λ*_*NO*_ in *Ω*_*NO*_. In practice, each model is evolved over the entire image, but after convergence restricted to either *Ω*_*O*_ or *Ω*_*NO*_, as initially defined by the coherence threshold. This avoids any noticeable artifacts at the boundaries of the regions by using the image itself to effectively pad each region for its model of diffusion along internal interfaces.

We call the decomposition model defined by the PDE of Eq. 14, the Oriented Laplacian Osher-Solé-Vese (OLOSV) decomposition model.

## Denoising by mean curvature motion

A similar denoising model to the OLOSV model developed in Eq. 14 above is the mean curvature motion (MCM) denoising model. Both models are based on the idea of denoising edges, only along and not across them. However, it will be seen that MCM denoising does not lend itself readily to a dual norm formulation, and thus cannot avail of the better quality results obtained from such a formulation.

The equation defining Mean Curvature Motion denoising is

15 $$ u_{t} = u_{\zeta\zeta} = |\nabla u|\text{div}\left(\frac{\nabla u}{|\nabla u|}\right),  $$

with *u*_*ζζ*_ the second-order directional derivative of *u* in the isophote direction.

To make a fair comparison with the OSV and OLOSV models in Eqs. 4 and 14, respectively, a fidelity term is added to the basic MCM of Eq. 15, along with a parameter *λ* that controls the relative weighting of the diffusion and fidelity terms. Thus the version of MCM implemented in this paper for comparison with OSV and OLOSV is

16 $$ u_{t} = \frac{1}{2\lambda}u_{\zeta\zeta} - (u-f).  $$

The implementation of mean curvature motion that we used in our experiments was based on the following

17 $$ u_{\zeta\zeta} = {\zeta_{x}^{2}} u_{xx} + 2\zeta_{x}\zeta_{y} u_{xy} + {\zeta_{y}^{2}} u_{yy},   $$

derived by setting (*ζ*_*x*_,*ζ*_*y*_)=(*f*_*iso*,*x*_,*f*_*iso*,*y*_) with this isophote direction measured in a noise-resistant manner, as in Section 2.2.1. We found from our experiments that using these image isophotes gave better denoising results.

### Explicit timestepping

Then Eq. 14 (for OLOSV) and Eq. 16 (for MCM) are each separately solved via explicit timestepping (with the time step chosen by experimentation to be *Δ* *t*=0.002).

As with other decomposition/denoising schemes, with known texture/noise variance, the value of *λ* is dynamically updated with iteration number using a method based on gradient projection. In fact, in oriented regions (*Ω*_*O*_) one dynamically updated coefficient *λ*_*O*_ is used, and in non-oriented regions, a separate dynamically updated coefficient *λ*_*NO*_ is calculated. The final formula that is obtained for *λ*_*O*_ is

18 $$ \lambda_{O} = \frac{1}{2\sigma^{2}}\int_{\Omega_{O}}(f-u)\left(c_{\zeta}K_{\zeta\zeta}+c_{\eta}K_{\eta\eta}\right)dxdy   $$

In non-oriented regions, the expression for *λ*_*NO*_ at each iteration is

19 $$ \lambda_{NO} = \frac{1}{2\sigma^{2}}\int_{\Omega_{NO}}\left(f-u\right)\Delta K(u) dxdy.  $$

The above two equations are derived from Eqs. 14 and 4, respectively, by assuming we have reached equilibrium, so that *u*_*t*_=0, and then multiplying each side by *u*−*f* and integrating over the image domain *Ω*.

For MCM denoising, a formula similar to Eq. 18 is used to determine *λ*_*O*_ at each iteration, also based on gradient projection

20 $$ \lambda_{O} = \frac{1}{2\sigma^{2}}\int_{\Omega_{O}}(f-u)u_{\zeta\zeta} dxdy.   $$

## Numerical implementation

Two sets of experiments were conducted using the OSV and OLOSV methods. The first involved an explicit timestepping solution of the two models, along with the mean curvature denoising model. The second set of experiments was based on the dual formulation of total variation minimization of Chambolle [12], which was further generalized in [13] to deal with the OSV model, and to an even more general TV-Hilbert model in [14]

21 $$ p_{i,j}^{n+1} = \frac{p_{i,j}^{n}+\Delta t\left(\nabla \left(K^{-1}\text{div}\left(p^{n}\right)-\lambda f\right)\right)_{i,j}}{1+\Delta t|\left(\nabla\left(K^{-1}\text{div}\left(p^{n}\right)-\lambda f\right)\right)_{i,j}|},  $$

with initial condition

$$p^{0} = 0. $$

Then, similar to [14], $f+\frac {1}{\lambda _{NO}}\Delta \text {div}p^{n}$ converges to the *u* component of the decomposition in *Ω*_*NO*_, the non-oriented section of the image, which implies that $-\frac {1}{\lambda _{NO}}\Delta \text {div}p^{n}$ converges to the component *v*=*f*−*u* in *Ω*_*NO*_. Similarly, in the oriented section *Ω*_*O*_ of the image, $f+\frac {1}{\lambda _{O}}\Delta _{\zeta \zeta }\text {div}p^{n}$ converges to the *u* component of the decomposition and thus $-\frac {1}{\lambda _{O}}\Delta _{\zeta \zeta }\text {div}p^{n}$ converges to the component *v*=*f*−*u* in *Ω*_*O*_.

When used in conjunction with the OLOSV model, such an implicit numerical method gives rise to a new denoising method, which we call the Implicit Oriented Laplacian Osher-Solé-Vese, or IOLOSV method.

## TV-Hilbert space formulation

The proposed IOLOSV model presented in this paper differs from the general TV-Hilbert model in [14] and the TV-Gabor model in [15], in that, in the proposed model, the operator *K* is spatially varying, whereas in the two other models, the operator *K* is constant throughout the entire image, and not data-dependent.

For two discrete zero-mean image functions *f* and *g*, Aujol and Gilboa [14] defined the inner product <·,·> on a Hilbert space $\mathcal {H}$ as

22 $$ <f,g>_{\mathcal{H}} = <f,Kg>_{L^{2}},   $$

where $<f,Kg>_{L^{2}} = \sum _{i,j} f_{i,j}(Kg)_{i,j}$. Then the Sobolev space *H* ^−1^ used in the model of Osher, Solé and Vese fits into the TV-Hilbert space framework, with the operator *K*=−*Δ* ^−1^ as the negative of the inverse Laplacian operator.

The proposed IOLOSV method can also be made to fit into the TV-Hilbert space decomposition framework, with the operator *K* spatially varying and equal to $K = -\Delta _{\zeta \zeta }^{-1}$, the negative of the spatially varying second-order directional derivative (in the direction of the image isophote). We denote the Hilbert space thus obtained as $H^{-1}_{\zeta }$ in order to emphasize its link with both the Sobolev space *H* ^−1^ and its dependence on the spatially varying isophote direction *ζ*(*x*,*y*).

**Fig. 2:**
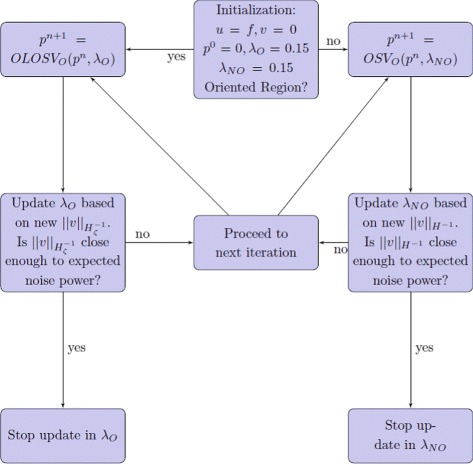
A flowchart illustrating the overall procedure for denoising with OLOSV and OSV models in the oriented and non-oriented parts of the image, respectively

A flow chart is included below illustrating the overall procedure for denoising with the OLOSV and OSV models in the oriented and non-oriented parts of the image, respectively (Fig. 2).

### Calculation of iteration-dependent fidelity parameter

It is shown in [14] that for an *M*×*N* pixel image *n* consisting only of white Gaussian noise of standard deviation *σ*, it can be assumed that

23 $$ ||n||^{2}_{\mathcal{H}} = C_{\mathcal{H}}||n||^{2}_{L^{2}} = C_{\mathcal{H}}MN\sigma^{2},  $$

where $C_{\mathcal {H}}$ is a constant that only depends on the Hilbert space $\mathcal {H}$ over which we are taking the norm of the noise image *n*. Its calculation will be described in the next subsection.

The same procedure found in [14] is used in this paper to dynamically vary the fidelity parameters *λ*_*O*_ and *λ*_*NO*_. This procedure is based on attempting to solve the problem

24 $$ \min_{u}\left\{\int_{\Omega}|\nabla u| dxdy\right\} \text{such that} ||u-f||_{\mathcal{H}}^{2} = C_{\mathcal{H}}MN\sigma^{2}.  $$

In practice, it was found in [14] that the problem posed by Eq. 24 leads to the oversmoothing of *u*; instead a positive factor $\alpha _{\mathcal {H}} < 1$ was added in [14] to the right-hand side of that equation to form the new problem

25 $$ {}\min_{u}\left\{\int_{\Omega}|\nabla u| dxdy\right\} \text{such that} ||u-f||_{\mathcal{H}}^{2} = \alpha_{\mathcal{H}} C_{\mathcal{H}}MN\sigma^{2}.  $$

In both equations, the noise component *v* is defined to be equal to *f*−*u*, where *f* is the original noise-contaminated image and *u* is the obtained cartoon component.

Based on our experimentation with the IOLOSV coefficients, both *λ*_*O*_ and *λ*_*NO*_ are initialized to 0.15, and then at each iteration, their values are updated using the following method. At every iteration, $||v_{O,n}||_{H^{-1}_{\zeta }}\phantom {\dot {i}\!}$ and $||v_{NO,n}||_{H^{-1}}\phantom {\dot {i}\!}$ are estimated by calculating $||v||_{L^{2}}\phantom {\dot {i}\!}$, easily done by taking the square-root of the sum of squares of the *v* component of the decomposition, and then multiplying by the factor $\sqrt {C_{H^{-1}}}\phantom {\dot {i}\!}$ for the non-oriented section of the image, and $\sqrt {C_{H_{\zeta }^{-1}}}\phantom {\dot {i}\!}$ for the oriented part. As in [14], given *λ*_*n*_ and $||v_{n}||_{\mathcal {H}}\phantom {\dot {i}\!}$, *λ*_*n*+1_ is obtained from these quantities by the formula

26 $$ {\begin{aligned} \lambda_{n+1} = \frac{||v_{n}||_{\mathcal{H}}}{\sqrt{\alpha_{\mathcal{H}} C_{\mathcal{H}}}MN\sigma}\lambda_{n}. \end{aligned}}  $$

In the above equation, $||v_{n}||_{\mathcal {H}}$ is the norm of *v*_*n*_ in the Hilbert space $\mathcal {H}$; the calculation of this norm is detailed below in Section 5.3. Then *v* is calculated using this new value of *λ* for the next iteration. The procedure is implemented separately in *Ω*_*O*_, where $\mathcal {H} = H^{-1}_{\zeta }$, and in *Ω*_*NO*_, where $\mathcal {H} = H^{-1}$. Moreover, the constant $\alpha _{\mathcal {H}}$ is chosen separately in each of *Ω*_*O*_ and *Ω*_*NO*_, with the selected values being 0.6 and 0.98, respectively, based on experimental findings.

For *Ω*_*O*_ we slightly alter the calculation of the numerator of Eq. 26 to ensure enough noise is extracted from oriented parts of the image. We calculate the *L* ^2^-norm of the noise in the non-oriented region *Ω*_*NO*_ and then compute the expected *L* ^2^-norm of the noise in the oriented region *Ω*_*O*_ by balancing the value of this norm in *Ω*_*O*_ and the sizes of both regions, by assuming that the noise power is spatially invariant across both regions. Finally, in the denominator of Eq. 26, we use the maximum of the actual measured *L* ^2^-norm of the noise in *Ω*_*O*_ and 0.7 times the expected *L* ^2^-norm of this noise in this region, based on that in *Ω*_*NO*_.

Execution of the proposed algorithm is stopped when the numerator and denominator of the multiplicative factor in Eq. 26 become close to one another. More specifically, for the non-oriented region *Ω*_*NO*_, iterations are stopped once the numerator becomes greater than 0.95 times the denominator, while for the oriented region *Ω*_*O*_ we stop iterating once the numerator becomes greater than 0.99 of the denominator.

### Calculation of constant $C_{\mathcal {H}}$

In [13] it is shown that when $\mathcal {H} = H^{-1}$, as is the case for the OSV model,

27 $$ C_{H^{-1}} = \frac{1}{MN}\sum_{\substack{p=0,q=0\\(p,q)\neq(0,0)}}^{p=M,q=N}\frac{1}{2\left(2-\cos\left(\frac{2\pi}{M}p\right)-\cos\left(\frac{2\pi}{N}q\right)\right)}.  $$

We first observe that the denominator of the expression for $C_{H^{-1}}\phantom {\dot {i}\!}$ corresponds to the discretization of the Laplacian operator in the frequency domain. Therefore, similar to the *H* ^−1^ case and following the methodology in [13], a similar summation expression is obtained for $\phantom {\dot {i}\!}C_{H^{-1}_{\zeta }}$, with the denominator of the summation now corresponding to the discretization of the oriented Laplacian in the frequency domain instead of the discretization of the plain Laplacian in the frequency domain. Since this only corresponds to one directional derivative, instead of two orthogonal directional derivatives, as is the case with the usual Laplacian operator, the denominator will be half the value of that for the usual Laplacian. Therefore, we will have

28 $$ C_{H^{-1}_{\zeta}} = 2 C_{H^{-1}}.  $$

### Calculation of $\mathcal {H}$-norms

Recall from elementary Hilbert space theory that the norm of a function on a Hilbert space is defined as the square root of the inner product of the function with itself. From the inner product definition of Eq. 22, the square of the $\mathcal {H}$-norm of *v*, $||v||_{\mathcal {H}}$ is thus defined by

29 $$ ||v||^{2}_{\mathcal{H}} = <v,v>_{\mathcal{H}} = <v,Kv>_{L^{2}}  $$

In the case of the OSV model, where $\mathcal {H}=H^{-1}$, and *K*=−*Δ* ^−1^,

30 $$ ||v||^{2}_{H^{-1}} = <v,-\Delta^{-1}v>_{L^{2}}.  $$

This equation was shown in [13] to simplify, based on Parseval’s identity, to

31 $$ {{\begin{aligned} {}||v||^{2}_{H^{-1}} \,=\, \frac{1}{MN}\!\sum_{\substack{p=0,q=0\\ (p,q)\neq(0,0)}}^{p=M,q=N}\!\frac{1}{2\left(2-\cos\left(\frac{2\pi}{M}p\right)\,-\,\cos\left(\frac{2\pi}{N}q\right)\right)}\left(|\mathcal{F}(v)(p,q)|\right)^{2}, \end{aligned}}}  $$

with $\mathcal {F}(v)$ being the discrete Fourier transform of *v*, and the image assumed to be *M*x*N* pixels large. From Eqs. 23 and 28 above, we find that

32 $$ {\begin{aligned} ||v||^{2}_{H^{-1}_{\zeta}} = 2||v||^{2}_{H^{-1}}, \end{aligned}}  $$

both of these squared-norm values being used in the dynamic update of the fidelity parameter *λ*, for their respective Hilbert spaces.

## Non-local means

To set this paper within the forefront of research on image denoising, we also compare the proposed OLOSV algorithm with non-local means, a recent addition to the field. Non-local means denoising, introduced in [16] by Buades et al. is known to give good denoising results. It does so by comparing image patches from different parts of an image and using all or many of these patches to improve denoising performance by performing a weighted average on these patches based on their similarity in pixel intensities.

From [16], the non-local (NL) mean of a function $u:\Omega \rightarrow \mathbb {R}$ is defined as

33 $$ \bar{u} := \frac{\int_{\Omega} u(y) \omega(x,y) dy}{\int_{\Omega} \omega(x,y) dy},  $$

where *ω*(*x*,*y*) is a weighting function measuring the similarity between the image patches centred at pixels *x* and *y*.

## Test images

Among the test images used in this paper are several well-known images with significant oriented texture, i.e. barbara which is 512 × 512 pixels, fingerprint which is 256 × 240 pixels, and bridges which is 1024 × 683 pixels, each with bitdepth of 8 bits.

Results obtained for the three test images barbara, bridges and fingerprint are presented in the next section; these results demonstrate that in general, denoised images with higher signal-to-noise ratios are obtained with IOLOSV over both the MCM and OSV algorithms, and the results are visually superior.

## Experimental results

### Experimental method

To each of the three original test images, additive white Gaussian noise (AWGN) was added with a standard deviation *σ*=15.

Then each of the four local iterative denoising algorithms (OSV, OLOSV, IOLOSV and MCM) was run on the noisy test images to obtain a denoised image; the signal-to-noise ratio is then calculated against the noise-free original image. We also ran the non-local means (NLM) algorithm on all three images. For non-local means, 5 × 5 pixel image patches were used to calculate the weight function measuring similarity between patches centred at each pixel in 11 × 11 neighborhoods.

Of the four iterative algorithms, only the proposed OLOSV model with implicit timestepping (IOLOSV) had a well-defined stopping condition, whereas the other 3 algorithms were run until a peak in the calculated signal-to-noise ratio (SNR) was detected. This procedure could actually artificially inflate the SNRs of the other 3 algorithms with respect to IOLOSV, since in practice, the SNR is not easily calculated for an image being denoised without having the denoised image at our disposal.

As already mentioned in the description of the proposed IOLOSV method in Section 5.1, the values of the fidelity parameter *λ* for the oriented and non-oriented regions of the image are updated dynamically for each of these regions. We stop the denoising process for the IOLOSV method separately for each of the oriented and non-oriented image regions, based on when the dynamic value of *λ* for each of these regions stabilizes.

### Results and discussion

Table 1 gives the SNRs of the denoised results from Osher-Solé-Vese decomposition (OSV), Oriented Laplacian OSV with explicit timestepping (OLOSV), Mean Curvature Motion (MCM), and Oriented Laplacian OSV with implicit timestepping (IOLOSV). As can be seen from that table, the NLM SNRs are generally the best, with the IOLOSV and MCM SNRs close behind.

**Table 1:** Final SNRs for local OSV denoising vs. OLOSV, MCM, IOLOSV and NLM denoising on test images

	Denoising method				
Image	OSV	OLOSV	MCM	IOLOSV	NLM
barbara	14.219	15.101	15.716	15.845	*17.313*
fingerprint	10.990	10.994	*11.963*	11.939	11.930
bridges	15.991	16.143	16.214	16.423	*16.834*

For each test image, the highest SNR value amongst the denoised results from each of the five algorithms tested is shown in italics

**Fig. 3:**
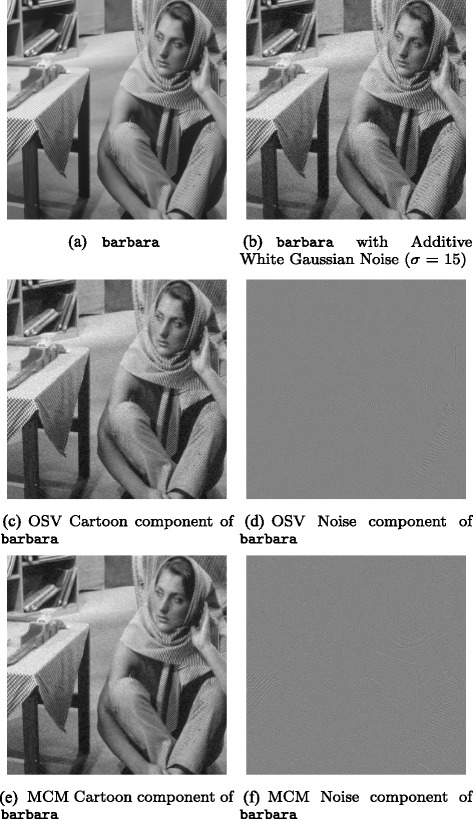
Original, noisy, OSV and MCM denoising results for barbara image. **a** barbara. **b** barbara with additive Gaussian noise (*σ*=15). **c** OSV cartoon component of barbara. **d** OSV noise component of barbara. **e** MCM cartoon component of barbara. **f** MCM noise component of barbara

**Fig. 4:**
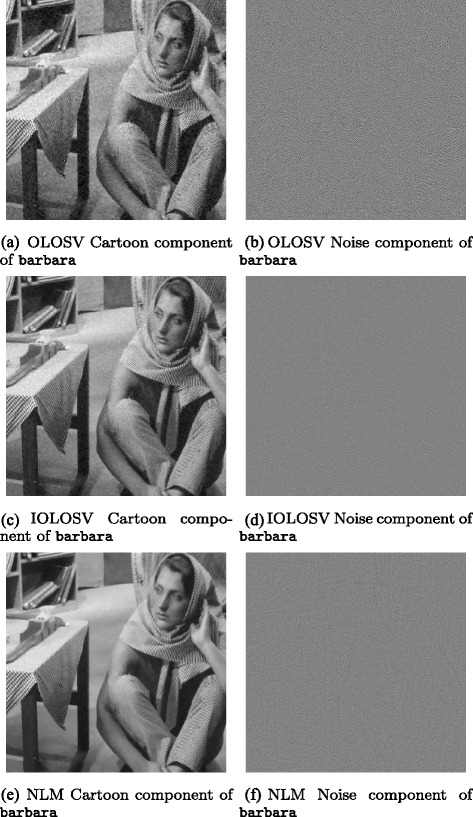
OLOSV, IOLOSV, and non-local means denoising results for barbara image. **a** OLOSV cartoon component of barbara. **b** OLOSV noise component of barbara. **c** IOLOSV cartoon component of barbara. **d** IOLOSV noise component of barbara. **e** NLM cartoon component of barbara. **f** MCM noise component of barbara

**Fig. 5:**
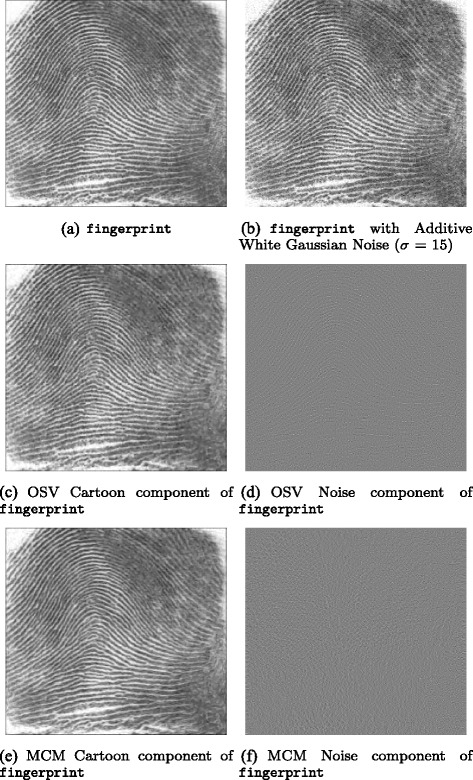
Original, noisy, OSV and MCM denoising results for fingerprint image. **a** fingerprint. **b** fingerprint with additive Gaussian noise (*σ*=15). **c** OSV cartoon component of fingerprint. **d** OSV noise component of fingerprint. **e** MCM cartoon component of fingerprint. **f** MCM noise component of fingerprint

**Fig. 6:**
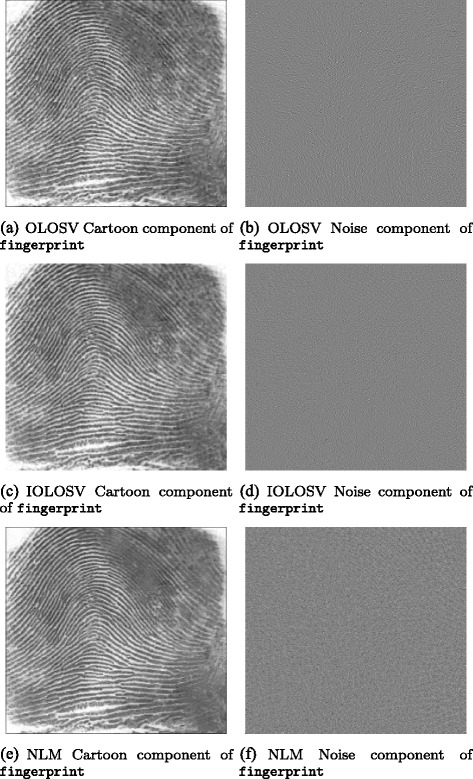
OLOSV, IOLOSV, and non-local means denoising results for fingerprint image. **a** OLOSV cartoon component of fingerprint. **b** OLOSV noise component of fingerprint. **c** IOLOSV cartoon component of fingerprint. **d** IOLOSV noise component of fingerprint. **e** NLM cartoon component of fingerprint. **f** NLM noise component of fingerprint

**Fig. 7:**
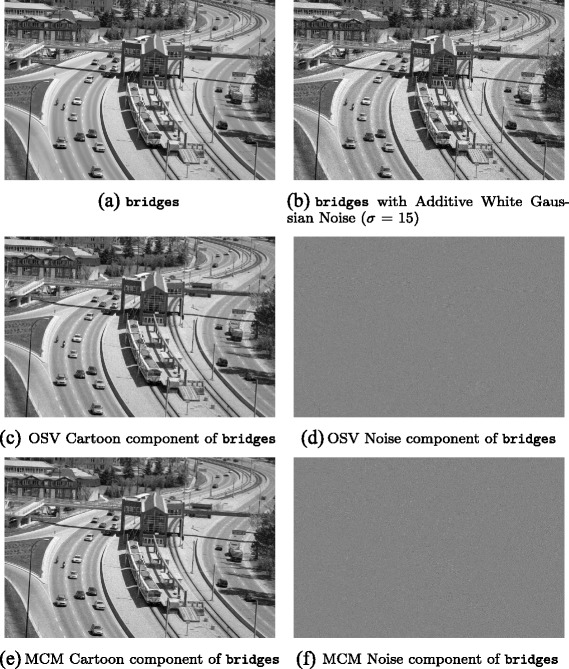
Original, noisy, OSV and MCM denoising results for bridges image. **a** bridges. **b** bridges with additive Gaussian noise (*σ*=15). **c** OSV cartoon component of bridges. **d** OSV noise component of bridges. **e** MCM cartoon component of bridges. **f** MCM noise component of bridges

**Fig. 8:**
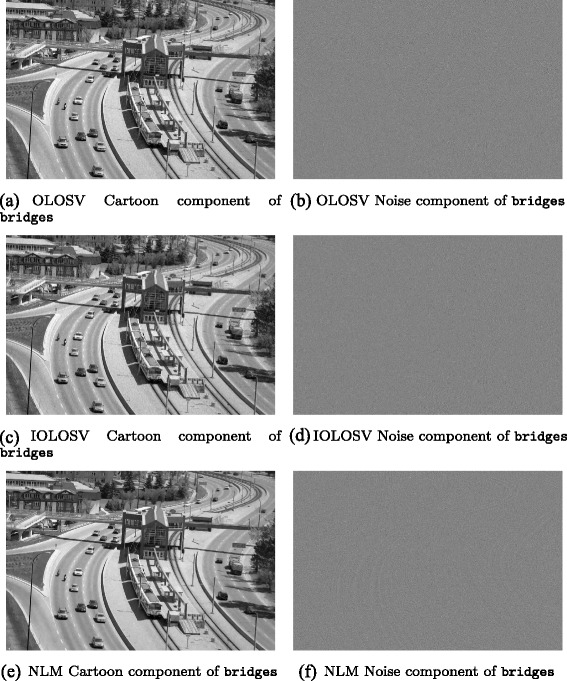
OLOSV, IOLOSV, and non-local means denoising results for bridges image. **a** OLOSV cartoon component of bridges. **b** OLOSV noise component of bridges. **c** IOLOSV cartoon component of bridges. **d** IOLOSV noise component of bridges. **e** NLM cartoon component of bridges. **f** NLM noise component of bridges

Figures 3, 4, 5, 6, 7 and 8 show the cartoon and noise components obtained from the various denoising algorithms on the three test images, along with the original and noisy images used for testing all of these methods. The appearance of the *v* component is much less ordered in the OLOSV and IOLOSV results than the results from the other algorithms. Despite the good SNR of NLM, it must be remembered that SNR is a global measure, which does not always accurately capture visual image quality. The noise components for all images processed with NLM exhibit very visible structure from the underlying noiseless image; this is not to be expected in typical white noise, indicating that the denoising results from NLM are not as good as the signal-to-noise ratio would suggest, and not as good as the results from the iterative variational methods.

**Fig. 9:**
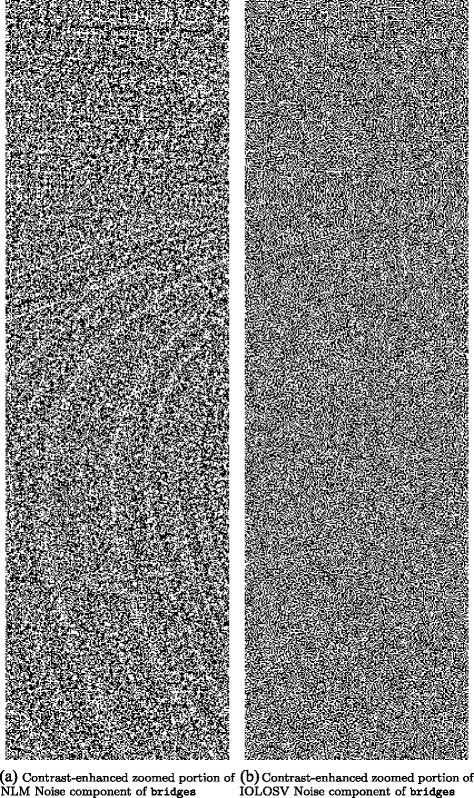
Comparison of contrast-enhanced and zoomed portion of noise components from **a** NLM and **b** IOLOSV models

This is further illustrated in Fig. 9, which depicts a zoomed and contrast-enhanced portion of the noise component obtained from the proposed IOLOSV method and the NLM algorithm. The contrast enhancement is done using the imadjust command found in Matlab^®;^’s Image Processing Toolbox. In the left panel, the NLM result contains visible structure from the original image which has been removed as part of the noise component, whereas in the right panel, corresponding to the same region from the IOLOSV result, such structures are not visible. Additionally, the NLM result takes longer to generate than the IOLOSV one, due to the high complexity of the non-local means algorithm.

## Conclusions

The OLOSV decomposition scheme, in both its explicit and implicit forms of implementation, has been found to be extremely good at denoising oriented texture, and is a substantial improvement over the model of Osher, Solé and Vese on which it is based. It also has some other good properties.

For example, one good property of OLOSV is that it can be generalized to images where there are up to two dominant isophote orientations at a point. These orientations can be calculated using the multiple orientation framework in [17]. In regions where there are two orientations present, the model’s evolution equation consists of the sum of two oriented Laplacians with isophote directions *ζ*_1_ and *ζ*_2_ and gradient directions *η*_1_ and *η*_2_.

Additionally, it was found from the experiments in this paper that cartoon edges were much less visible in the noise components *v* of the OLOSV and IOLOSV results when compared with the OSV noise components (e.g. compare Fig. 5 d to Fig. 6 b, d). This is because a directional diffusion was performed close to these cartoon edges (if they were calculated as being in the coherent regions of the image), and along the direction of the edges, and not perpendicular to them. Unfortunately, there tends to be some slight visible smearing of the noise close to the edges, but this could be reduced by only including pixels *on* cartoon edges in the region in the image computed to have a coherent orientation, and not those around the edges. The detection of such cartoon edges could be implemented with a Total-Variation-like diffusion, similar to [18].

It may be argued that the Oriented Laplacian OSV model used for denoising is very similar to ordinary Oriented Laplacian diffusion, which when the diffusion across edges is set to zero, degenerates to the mean curvature motion denoising model [19] tested in this paper. However, the OLOSV model has two main differences with standard Oriented Laplacian diffusion:

1. Mathematically OLOSV is a novel nonlinear diffusion flow based on the Laplacian of the level-set curvature of the cartoon component, instead of the Laplacian of the image itself, and
2. OLOSV is based on a model for image decomposition, and thus variations of it could potentially be used for other applications of image decomposition, e.g. inpainting.

In addition to the above two distinguishing properties of the proposed OLOSV model with respect to mean curvature motion, OLOSV also has two distinct advantages:

1. The proposed OLOSV model fits into the Hilbert space formulation of [14], and therefore admits an implicit timestepping implementation, which we called IOLOSV in this paper, as opposed to mean curvature motion which does not. This means that the proposed method is far more efficient; this is confirmed by our experiments, that required only on the order of ten iterations for convergence, as opposed to several hundred for mean curvature motion.
2. The proposed IOLOSV method admits an easy-to-define stopping criterion which allows automatic cessation of execution of the algorithm when the SNR is at or close to its peak value. Such a stopping criterion is not as easily defined for mean curvature motion, necessitating human intervention to determine when the image has in fact been denoised.

Finally, the OLOSV decomposition model can be extended quite easily to colour images by using the colour total variation model of Chan and Blomgren [20]. This remains as future work.
